# Habitat selection and refuge‐use by a color polymorphic salamander reveal behavioral niche differences

**DOI:** 10.1002/ece3.10978

**Published:** 2024-02-26

**Authors:** Cory S. Straub, Rosella G. Cuomo, Gabriel Jimenez

**Affiliations:** ^1^ Department of Biology Ursinus College Collegeville Pennsylvania USA

**Keywords:** behavior, divergent selection, *Plethodon cinereus*, thermal niche

## Abstract

Color polymorphic species provide an excellent opportunity to investigate the ecology and evolution of intraspecific niche differences. The red‐backed salamander, *Plethodon cinereus*, is a fully terrestrial lungless salamander with two common color forms, striped and unstriped. Previous research suggests the morphs may be differentially adapted to surface and subsurface microhabitats, with the unstriped morph being more fossorial. This hypothesis predicts that the unstriped morph should be more sensitive to the risks of surface activity (e.g., thermal stress, dehydration, predation), and therefore be more selective than striped morphs when choosing soil surface microhabitats. To test this hypothesis, we experimentally manipulated leaf litter mass in small forest patches (~0.45 m^2^). Leaf litter addition reduced soil temperatures, buffered against changes in air temperature, and likely provided physical protection from predators. Over 3 years, we found that unstriped adults responded positively to leaf litter addition, but striped adults did not. In addition, unstriped morphs spent significantly more time in protective refuges (opaque, moistened tubes) than striped morphs in laboratory assays. Taken together, the field and laboratory results support the hypothesis that the unstriped morph is more sensitive to the risks of surface activity, and therefore is more likely to be fossorial. This difference in microhabitat use, combined with spatiotemporal variation in leaf litter accumulation on the forest floor, may play an important role in the maintenance of the polymorphism.

## Graphical abstract

The red‐backed salamander has two common color forms, striped and unstriped. In this study, the unstriped morph showed a stronger response to surface habitats with leaf litter addition, and they showed higher refuge‐use in the laboratory. These behavioral differences are consistent with the view that the unstriped morph is more fossorial, and this niche difference combined with spatiotemporal variation in leaf litter accumulation on the forest floor may contribute to the maintenance of the polymorphism.

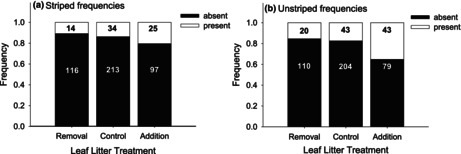

## 1 INTRODUCTION

Color polymorphisms, in which two or more distinct color phenotypes that are genetically based coexist within an interbreeding population, have attracted the attention biologists for decades (Bond, 2007; Cain & Sheppard, 1954; Endler et al., 1997; Ford, 1945). In addition to their esthetic appeal, color polymorphic species are ideal for study because changes in color morph frequencies across space and time are easy to detect and can yield insight into the ecology and evolution of such species. Understanding the microevolutionary forces that promote the long‐term persistence of color polymorphisms, the role of color polymorphism in the generation of new species (Gray & McKinnon, 2007; Jamie & Meier, 2020; McLean & Stuart‐Fox, 2014), and the consequences of color polymorphism for populations (e.g., range size and extinction risk), remain important challenges in evolutionary ecology (Forsman, 2016).

At least eight species of forest dwelling salamanders in the family Plethodontidae show a dorsal‐stripe polymorphism, with individuals being striped or unstriped (Fisher‐Reid & Wiens, 2015; Fitzpatrick et al., 2009; Highton, 2004). The Eastern red‐backed salamander, *Plethodon cinereus*, displays this polymorphism and is the most abundant and well‐studied salamander in eastern North America (Jaeger et al., 2016; Moore & Ouellet, 2015). The stripe polymorphism in this species is heritable and involves only a few genetic loci (Highton, 1959, 1975). Some plasticity in response to temperature could occur at the egg stage (Evans et al., 2020), but after hatching the dorsal stripe is a fixed trait. The frequency of the striped morph within populations varies dramatically (0%–100%) across the species range (Cosentino et al., 2017; Moore & Ouellet, 2015), with an average striped frequency of ~70% (Fisher‐Reid & Wiens, 2015). A combination of selection, drift, and gene flow are thought to work together to maintain the dorsal‐stripe polymorphism in this species (Hantak et al., 2019), but an understanding of how differential selection operates within and across populations remains unclear.

Forsman et al. (2008) developed a model to predict the ecological and evolutionary consequences of color polymorphism (Forsman et al., 2008). In this model, once a color polymorphism evolves it becomes coupled with other traits to create “ecomorphs” that occupy different niches. Red‐backed salamanders regularly move between surface and subsurface microhabitats (Taub, 1961), and several lines of evidence suggest that striped morphs spend more time on the soil surface than unstriped morphs. In the field, striped morphs occupy their soil surface territories for longer periods of time than unstriped morphs, and are recaptured there more frequently (Reiter et al., 2014). In the laboratory, when striped and unstriped morphs are placed together in three dimensional microcosms, striped morphs spend more time on the soil surface and the unstriped morphs spend more time in subsurface refuges (Dallalio, 2013). Finally, a study of museum specimens collected over 43‐years showed that striped morphs have become smaller with increasing temperature (Hantak et al., 2021), which is the expected response to a warming climate (Peralta‐Maraver & Rezende, 2021). In contrast, unstriped morphs showed little change in body size, perhaps because they have largely avoided warming by using subsurface refugia.

The striped and unstriped morphs have morphological features that may reflect differential adaptation to surface and subsurface lifestyles. Using models of avian, snake, and mammal visual systems, Hantak and Kuchta (2018) found that under most field conditions striped morphs are better camouflaged than unstriped morphs on the soil surface. Thus, the dorsal stripe might be an adaptation that evolved to reduce detection by surface‐active predators. Indeed, several studies have found a higher incidence of tail breakage in unstriped morphs, perhaps because they are more easily detected by visually hunting predators (Moreno, 1989; Venesky & Anthony, 2007). Striped and unstriped morphs also differ in costal groove number (range 18–20), and the unstriped morph tends to have more costal grooves than the striped morph (Fisher‐Reid et al., 2013; Williams et al., 1968). Importantly, the elongated trunk that results from more costal grooves is thought to facilitate burrowing and is associated with greater fossoriality in plethodontids (Jockusch, 1997; Parra‐Olea & Wake, 2001; Wake, 1964).

Striped and unstriped red‐backed salamanders also differ in physiological adaptations that may reflect differences in surface activity. Surface activity increases salamanders' exposure to predators and while both color morphs respond to predator attacks by autotomizing their tails, the detached tails of striped morphs move faster and longer, making them more effective distractors (Otaibi et al., 2017). Increased surface activity also increases salamanders' risk of dehydration and while the morphs lose water at similar rates, the striped morph has been shown to rehydrate more quickly (Smith et al., 2015). These physiological differences between the color morphs are consistent with the view that striped morph spends more time on the soil surface.

Taken together, the available evidence suggests that the unstriped morph may function as a soil surface specialist, primarily occupying this microhabitat under ideal conditions when the risks of surface activity are low. In contrast, the striped morph may be a soil surface generalist, occupying this microhabitat across a broader range of conditions when the risks of surface activity are both low and high. Leaf litter on the forest floor benefits red‐backed salamanders (Maerz et al., 2009; Ziemba et al., 2016) and can act as a physical buffer against warm and dry conditions, and it may conceal salamanders from visually hunting predators. In this study, we experimentally manipulated leaf litter mass in small forest patches to test the hypothesis that the unstriped morph would be more sensitive to variation in leaf litter mass. We also conducted a behavioral assay in the laboratory, in which striped and unstriped morphs were placed in an arena with a physical refuge (an opaque, moistened tube). We predicted that, compared with the striped morph, the unstriped morph would respond more strongly to leaf litter addition in the field, and would spend more time in protective refuges in the laboratory, because of their greater sensitivity to thermal stress, dehydration, and/or predation risk.

## 2 MATERIALS AND METHODS

### 2.1 Ethics statement

The field and laboratory studies were conducted with prior approval of the Institutional Animal Care and Use Committee at Ursinus College (field IACUC protocol #6010‐02, lab IACUC protocol #6012‐01). Animals for the lab study were collected with the Pennsylvania Fish and Boat Commission scientific collecting permit 2021‐01‐CS Type 1.

### 2.2 Habitat selection

#### 2.2.1 Field study site and design

The study took place in a forested area on the Ursinus College campus in Montgomery County, Pennsylvania (40.195555, −75.451914). The deciduous forest includes a mix of *Carya ovata*, *C. cordiformis*, *Acer saccharum*, *A. negundo*, *Fraxinus americana*, *Prunus serotina*, *Ulmus rubra*, *Quercus rubra*, and *Q. alba*. Soil type includes a mix of Penn silt loam and Penn Klinesville channery silt loam. The forest slopes down (~15% slope) to the nearby Perkiomen creek.

In fall 2016, we set up an array of artificial cover objects (ACOs) on the forest floor. ACOs were made of fir plywood cut to 30 × 30 × 1.9 cm. ACOs were placed along 23 transects running West‐to‐East and downslope. Transects included eight ACOs (*n* = 184). The 23 transects were separated from each other by 5 m, and the eight ACOs within each transect were separated from each other by 5 m. The highest‐elevation ACO was 58 m, and the lowest‐elevation ACO was 43 m above sea level.

**FIGURE 1:**
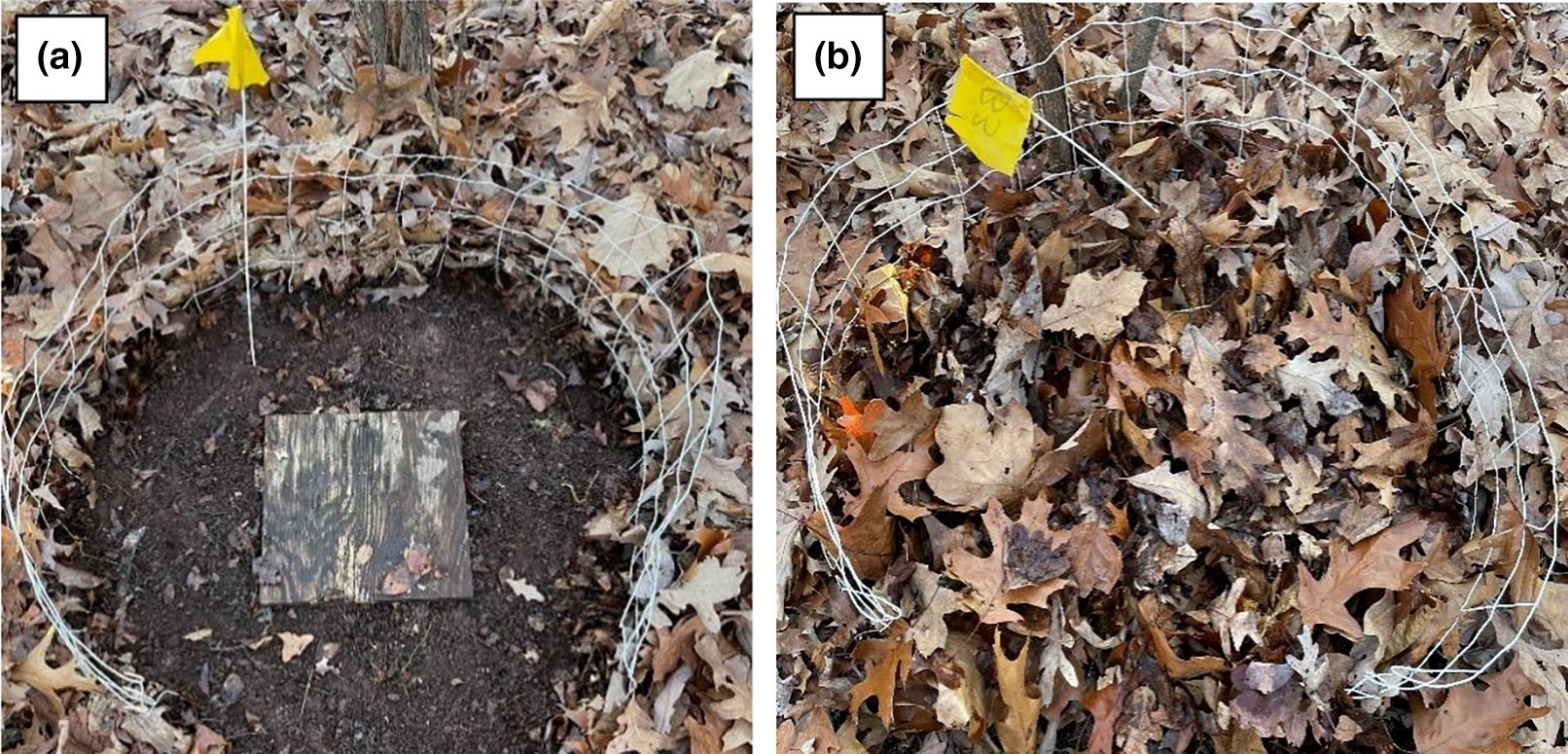
Plots showing the leaf litter removal (a) and addition (b) treatments. The artificial cover object (ACO) is present in both treatments but is only visible in (a).

In spring 2017, methods for salamander capture and measurement, and for the measurement of soil temperature and moisture, were developed and refined. The leaf litter manipulation experiment was initiated in spring 2018. Three treatments were created – leaf litter addition, removal, and control. In the leaf litter addition and removal treatments, wire garden fencing (~36 cm height) was placed in a circle (~0.76 m diameter) surrounding the ACO, with ~36 cm on the downhill side open to allow access to the ACO when sampling (Figure 1). The fencing served to keep leaf litter in and out of the addition and removal treatments, respectively. Within each transect, the 8 ACOs were randomly assigned to two addition, two removal, and four control treatments in which leaf litter was left unmanipulated. Across the entire array, this yielded a sample size of 46 for the addition and removal treatments, and 92 for the control treatment. However, treefall, failure to find control plots (due to lack of visible fencing), damaged fences from deer, and cases where the salamanders escaped before they could be collected resulted in the loss of some replicates. The experiment was conducted in 2018, 2019, and 2021, and the leaf litter treatment was re‐randomized and reestablished each year to avoid pseudo‐replication (Hurlbert, 1984). Leaf litter manipulations were made on March 21, 2018, March 20, 2019, and March 17, 2021. Leaf litter was collected from the forest floor and each leaf litter addition ACO received two 2‐gallon plastic bags of leaf litter. The dry mass of leaves added and removed was measured in six replicates per treatment in each year, with minor variation among years. From these data, we estimate that dry mass of leaves in the leaf litter addition, control, and removal plots was 588 g, 220 g, and negligible, respectively (Figure 1).

#### 2.2.2 Field sampling

Plots were sampled on six dates, two each year: April 11, 2018, April 18, 2018, April 10, 2019, April 17, 2019, April 7, 2021, and April 14, 2021. Each ACO was sampled only once per year, so recaptures within years were highly unlikely. Students in C. Straub's Animal Behavior laboratory conducted the sampling. Soil measurements were recorded from a location within 4 cm of the ACO. Temperature was recorded by placing an infrared thermometer (model: Oakton WD‐39642‐01) 1 cm above the soil surface, and moisture was recorded 8 cm below soil surface using a soil moisture probe (Soil PH & Moisture meter, ZD‐06; Gain Express Holdings, Ltd). ACOs were checked for salamanders. When present, the salamanders were placed individually in a small plastic bag and transported to a processing table in the field where salamander morph, age, and sex were recorded. Salamanders with a snout‐to‐vent length (SVL) ≥35 mm were considered adults (Sayler, 1966). Sex was determined for adults using the candling method (Gillette & Peterson, 2001) combined with the presence/absence of swollen nasolabial grooves and cirri that are indicative of mature males. After processing, salamanders were immediately returned to the ACO from which they were collected.

### 2.3 Refuge‐use

#### 2.3.1 Animal collection and care

Thirty‐six similarly sized adult males, 18 striped and 18 unstriped, were collected from the Ursinus College forest on October 14, 2021. We used males to reduce variation in the reproductive condition of the animals – females tend to reproduce every 2 years (Anthony & Pfingsten,^2013^) and we observed that some were gravid while others were not. Salamanders were housed in clear plastic containers (21.6 cm length, 13.3 cm width, 3.8 cm height) with a 50 mL falcon tube lined with a moistened unbleached paper towel to provide a refuge. Containers and tubes were cleaned and paper towels replaced every 2 weeks. Salamanders were fed 20–30 wingless *Drosophila melanogaster* each week. Salamanders were housed in an environmental chamber with low light and maintained at 12°C with a 12:12 L:D photoperiod. This housing temperature was slightly higher than the 10°C recommended by Jaeger (1992) for the long‐term care of this species. Red‐backed salamanders are mostly active at night, so the light cycle was reversed to observe their behavior during the day (11 am–11 pm dark period). Salamanders were acclimated to the reversed photoperiod for 3 months before the assay.

#### 2.3.2 Refuge‐use assays

For the behavioral assay, salamanders were moved from the environmental chamber to a testing room where their refuge‐use could be visually monitored. Temperature in the testing room was 19–21°C. The containers were arranged in a linear array with two rows. At each location along the array, a size‐matched pair of striped/unstriped salamanders was placed, and their relative position (row closer or further from the observer) was randomized and unknown to observers. Salamanders were moved at 10 am during the light period when most were in their refuges (light causes them to retreat to the tubes). At 11 am the lights were turned off in the testing room, and salamanders were given 1 h to acclimate to the new conditions before the trial began. Salamanders were observed every 30 min from 12 to 4 pm. Within the containers, the refuge tubes were positioned in the upper left corner with the opening facing the observer. Observers used a red headlamp to see the salamanders in the dark room and scored each salamander's location relative to the refuge as “in”, “partially out”, or “out”. Salamanders were considered partially out if their heads were out of the refuge. Preliminary analyses revealed that the morph more likely to be “partially out” was also more likely to be “out”, so these two behavioral categories were lumped into a single “out” category to increase the number of observations and improve the fit of the statistical model used in the analysis. The trial was repeated four times, twice a week with 1 day in‐between trials for 2 weeks. Observations were not made on two occasions, resulting in a total of 34 observations for each salamander. Salamanders were returned to the environmental chamber (12°C) in between trials. They were maintained on their normal feeding schedule (once per week) during the two‐week experimental period but were not fed on assay days.

To investigate the effects of morph and refuge‐use on foraging success, a feeding assay was conducted. The time of day, acclimation period, and spatial array of the containers was the same as described above, but 10 wingless *D. melanogaster* were introduced to the container by blowing them into a small hole in the containers. Tape was used to close the holes. Salamanders were given 1 h to feed, and the remaining flies were counted. The feeding assay was conducted in the week following the behavioral assay and two trials were run with 1 day in‐between them. The proportion of flies consumed (out of 20) was used in the analysis.

### 2.4 Statistical analyses

For analyses, we used the R‐workflow template (and packages therein) for generalized linear mixed model analysis (glmmTMB) provided by Santon et al. (2023). The template was used to select the most appropriate models by evaluating residual dispersion, zero inflation, and the overall model fits provided by different residual distribution families and link functions. All analyses were performed using R Statistical Software (*v*4.2; R Core Team, 2023).

#### 2.4.1 Habitat selection

##### Habitat selection: Morph analyses using presence/absence data

In the analysis of habitat selection in the field, striped and unstriped salamanders were analyzed separately. We focused our analyses on adult salamanders, and ACOs where a mixed‐morph male–female pair was found were omitted because in these cases it is unclear whether the morph was selecting the ACO based on the leaf litter treatment or the presence of the opposite sex. We first used Pearson Chi‐Square analysis of aggregated data from all 3 years of study to determine whether salamander presence varied across the three leaf litter treatments for striped and unstriped morphs separately. Morphs were also analyzed separately in the glmm analysis. For these analyses, the dependent variable was salamander presence/absence, and the independent variables were the leaf litter treatment (removal, control, addition), soil temperature, and soil moisture. ACO location was included in the models as a random effect. Variance inflation factors (VIFs) were calculated to assess whether multicollinearity among the independent variables was problematic. For both the striped and unstriped glmm analyses, a binomial distribution with the logit link function was used, and pairwise differences between leaf litter treatments are reported as an odds ratio with 95% compatibility intervals. These analyses were repeated on a restricted data set where salamander presence was limited to cases where only a single individual was present.

##### Habitat selection: Leaf litter effects on soil temperature and moisture using all ACOs

To investigate leaf litter effects on soil temperature and moisture, a dataset with all ACOs, regardless of salamander presence/number, was analyzed. Glmms with soil temperature and moisture as dependent variables, leaf litter treatment as the independent variable, and ACO location as a random effect were used. For both responses, the gaussian error distribution with the identity link function was appropriate. In addition, simple linear regression was performed to evaluate the relationship between air temperature and soil temperature for each leaf litter treatment separately using the lme4 package in R. In all cases, results from Wald type II chi‐squared tests were consistent with results of effect size tests (e.g., response odds ratios with 95% compatibility intervals), and we report the former here.

##### Habitat selection: Comparison of selected microhabitats

A data set restricted to ACOs where salamanders were present (i.e., microhabitats that were selected by salamanders) was analyzed to further explore the potential for morph differences in habitat selection. Separate glmms with soil temperature and soil moisture as dependent variables were used. Independent variables were morph and leaf litter treatment, and their interaction, and ACO location was modeled as a random effect. VIFs were calculated to determine if collinearity between the independent variables was problematic. These analyses were conducted on three datasets, one (“all”) where every salamander was included as an independent observation (even if they co‐occurred under the same ACO), one (“no mixed morphs”) where salamanders co‐occurring with the opposite morph of a different sex were excluded, and one (“singles”) where only single salamanders were present under the ACO. This resulted in six analyses. For the temperature analyses, a gamma error distribution with the log link was used for the “all” and “no mixed morphs” datasets. A gaussian error distribution with the identity link function was used in the analysis of the other four data sets.

##### Habitat selection: Testing for body size differences among leaf litter treatments and morphs

If leaf litter addition ACOs represent higher quality surface microhabitats, it is possible that the habitat selection results could reflect the result of competitive interactions, with larger salamanders controlling access to these sites. This hypothesis yields two predictions: (1) salamanders occupying the leaf litter addition microhabitats will be larger than those in the other leaf litter treatments, and (2) the color morph that is most often associated with the leaf litter addition treatment will be larger. To test these predictions, SVL was used as the dependent variable. A glmm with morph, leaf litter treatment, and their interaction as independent factors, and ACO location as a random effect was used. VIFs were calculated to determine if collinearity between the independent variables was problematic. These analyses were conducted on the three datasets described above, i.e., “all”, “no mixed morphs”, and “singles”.

##### Refuge‐use

The repeatability of refuge‐use behavior by individuals across trials was assessed by calculating the Pearson correlation coefficient for individuals in trial 1 (number of “in” refuge observations) and the sum of their “in” refuge observations across trials 2, 3, and 4. As with the habitat selection analyses, refuge‐use differences between striped and unstriped salamanders were analyzed using the glmmTMB package (R Core Team, 2023). In the initial analyses, the number of observations (in, out) served the dependent variables, and morph (striped, unstriped) and trial (1–4) were the independent variables. Morph × trial interaction terms were included in the models, and individual was entered as a random effect. Morph × trial interactions were not statistically significant (*p* > .05 for both the “In” and “Out” analyses), so trial was removed as a factor and the total number of observations summed across the four trials was used as the dependent variable. In these final analyses, each salamander served as a single independent replicate and so the random variable “individual” was not necessary. Glmms with the nbiom1 distribution family and log link function were used. In all cases, results from Wald type II chi‐squared tests were consistent with results of effect size tests and we report the former.

##### Refuge‐use: Foraging success

Anova was used to analyze the salamander foraging data using the *lm* function. The proportion of flies consumed (out of 20) was logit transformed to meet linear model assumptions, as recommended by Warton and Hui (2011). The effects of color morph, refuge‐use (i.e., number of “in” observations), and their interaction on foraging success were analyzed. To provide a second test of the effect of refuge‐use on foraging success, salamanders were divided into two equally sized groups: those that were observed in the refuge the most (*n* = 18; 28% striped) and those that were observed in the refuge the least (*n* = 18; 72% striped). Using this approach, the effect of refuge‐use (low vs. high) on foraging success was analyzed.

## 3 RESULTS

Over the 3 years of study, we observed 124 striped adults (40%) and 185 unstriped adults (60%), and morph frequency was consistent across years (2018: 39% striped; 2019: 44% striped; 2021: 40% striped). These salamanders were found underneath 198 different ACOs, and occurred as singles (62% of total), pairs (27% of total), triplets (8% of total), quadruplets (3% of total), and in one case each, there was five and seven individuals. When a striped morph occurred under an ACO, it was observed with at least one opposite sex individual 37.3% of the time, and with at least one same‐sex individual 32.4% of the time. When an unstriped morph occurred under an ACO, it was observed with at least one opposite sex individual 28.9% of the time, and with at least one same‐sex individual 31.1% of the time.

### 3.1 Habitat selection

#### 3.1.1 Habitat selection: Morph analyses using presence/absence data

**FIGURE 2:**
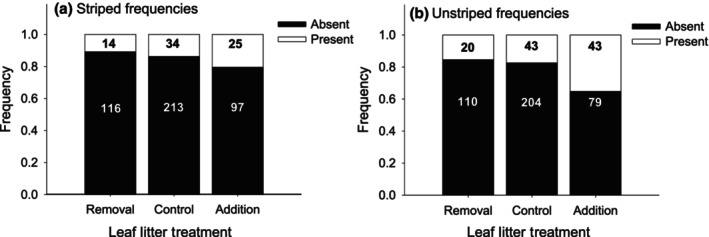
Presence/absence of striped (a) and unstriped adults (b) in the three leaf litter treatments. Numbers within the bars indicate the number of occurrences in each category. Data are from all 3 years of study.

Striped morphs were present under 10.8% of leaf litter removal ACOs, 13.8% of control ACOs, and 20.5% of addition ACOs. This difference was not significant (Pearson *χ* ^2^ = 5.06, df = 2, *p* = .08, Figure 2a). The glmm analysis revealed no effect of leaf litter treatment (Wald *χ* ^2^ = 2.04, df = 2, *p* = .361), soil temperature (Wald *χ* ^2^ = 0.67, df = 1, *p* = .412), or soil moisture (Wald *χ* ^2^ = 0.01, df = 1, *p* = .906) on striped morph presence probability. Restricting the dataset to single salamanders did not change this result (leaf litter: Wald *χ* ^2^ = 0.09, df = 2, *p* = .955; temperature: Wald *χ* ^2^ = 0.78, df = 1, *p* = .379; moisture: Wald *χ* ^2^ = 0.13, df = 1, *p* = .716). Multicollinearity was minimal – VIFs for leaf litter treatment, soil temperature, and soil moisture were all low (i.e., <1.2) and removing leaf litter treatment from the model did not substantially change the results for soil temperature and moisture.

Unstriped morphs were present under 15.4% of leaf litter removal ACOs, 17.4% of control ACOs, and 35.2% of addition ACOs. This difference was significant (Pearson *χ* ^2^ = 19.14, df = 2, *p* < .0001, Figure 2b). Glmm analysis revealed a significant effect of leaf litter treatment (Wald *χ* ^2^ = 13.25, df = 2, *p* = .001), but no effect of soil temperature (Wald *χ* ^2^ = 1.02, df = 1, *p* = .312) or soil moisture (Wald *χ* ^2^ = 0.31, df = 1, *p* = .577) on unstriped presence probability. Pairwise contrasts indicated that unstriped morphs occupied the leaf litter addition ACOs significantly more often than the control and the removal ACOs, but there was no difference between the control and removal ACOs (Table 1). Restricting the dataset to single salamanders yielded similar results (leaf litter: Wald *χ* ^2^ = 6.74, df = 2, *p* = .034; temperature: Wald *χ* ^2^ = 0.33, df = 1, *p* = .565; moisture: Wald *χ* ^2^ = 1.46, df = 1, *p* = .227).

**TABLE 1:** Pairwise contrasts from the glmm analysis of unstriped salamander response to the leaf litter treatments.

Data set	Contrast	Mean	Lower 95% CI	Upper 95% CI
ACOs with >1 salamander included	Removal/control	0.86	0.48	1.54
	**Removal/addition**	**0.33**	**0.18**	**0.62**
	**Control/addition**	**0.38**	**0.23**	**0.64**
ACOs with >1 salamander excluded	Removal/control	0.93	0.48	1.81
	**Removal/addition**	**0.44**	**0.21**	**0.93**
	**Control/addition**	**0.47**	**0.25**	**0.88**

*Note*: Mean pairwise response odds ratios with 95% CIs that do not overlap 1 (the null hypothesis) are bolded.

#### 3.1.2 Habitat selection: Leaf litter effects on soil temperature and moisture using all ACOs

**FIGURE 3:**
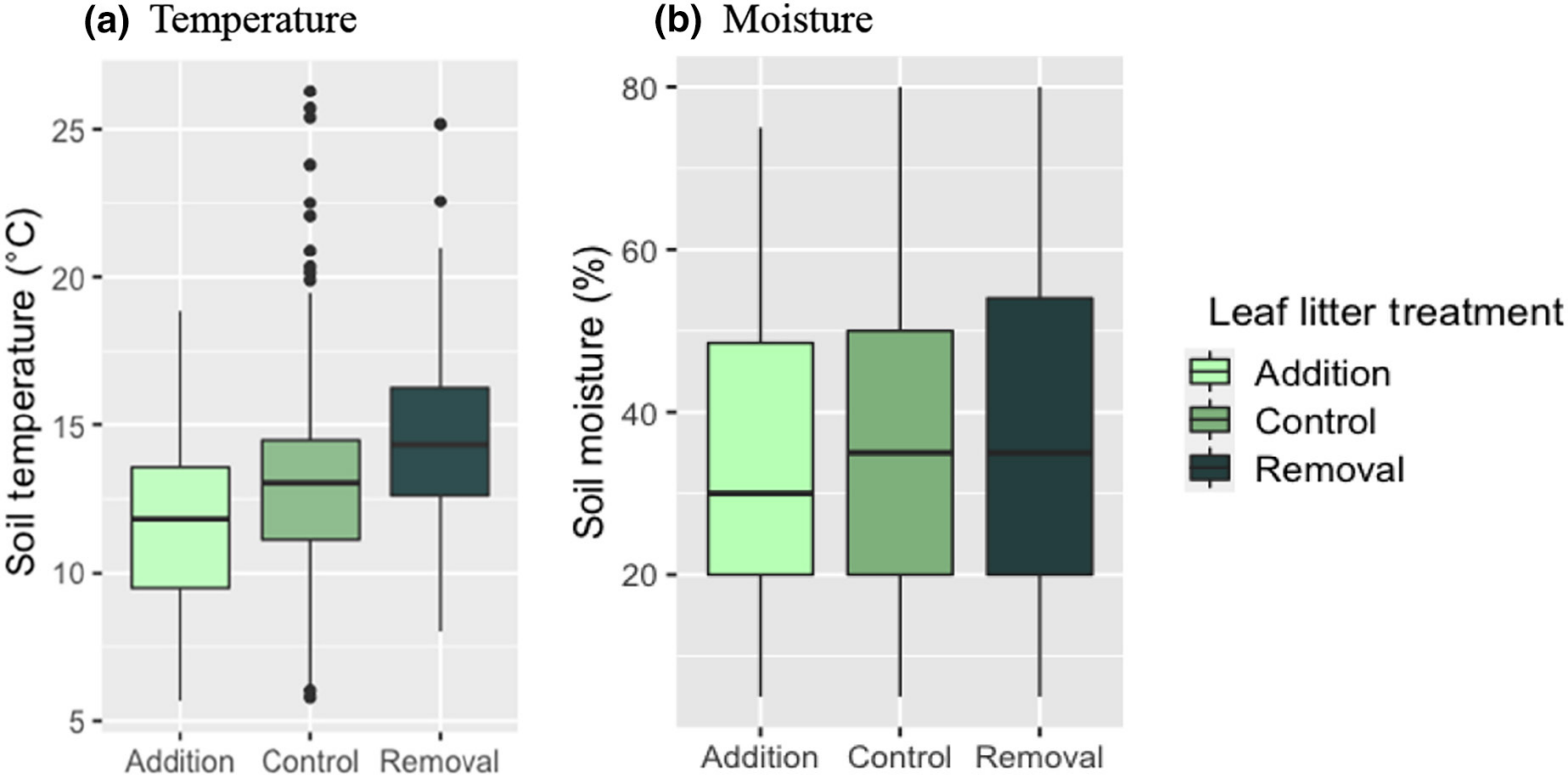
Leaf litter treatment effect on soil temperature (a) and moisture (b). In the box and whisker plots, the box shows the interquartile range with the median represented by the horizontal line. The whiskers show the first and fourth quartile, and outliers appear above and below the whiskers. There was a statistically significant effect of leaf litter on soil temperature, but no effect on soil moisture (see text for details).

Increasing leaf litter decreased soil temperature (means ± 1 SE: Removal: 14.7 ± 0.29°C; Control: 13.1 ± 0.2°C; Addition: 11.6 ± 0.30°C). This difference was significant (Wald *χ* ^2^ = 56.915, df = 2, *p* < .0001, Figure 3a). Leaf litter had little effect on soil moisture (means ± 1 SE: Removal: 36.1 ± 1.65%; Control: 36.6 ± 1.25%; Addition: 34.5 ± 1.68%). This difference was not statistically significant (Wald *χ* ^2^ = 1.173, df = 2, *p* = .556, Figure 3b). The leaf litter addition treatment provided a stronger buffer against changes in air temperature, as indicated by the lower slope and *R* ^2^ values for the relationship between air and soil temperature in this treatment (Removal: *β* = 0.35 ± 0.05, *p* < .0001, *R* ^2^ = .26; Control: *β* = 0.36 ± 0.04, *p* < .0001, *R* ^2^ = .22; Addition: *β* = 0.26 ± 0.06, *p* < .0001, *R* ^2^ = .13).

#### 3.1.3 Habitat selection: Comparison of selected microhabitats

Multicollinearity among the morph and leaf litter treatment variables was minimal – the VIF for both variables was 1 and removing leaf litter treatment from the models did not substantially change the results for soil temperature or moisture. Leaf litter effects on soil temperature and moisture were consistent with those shown in Figure 3, but there was no difference between morphs in soil temperature or moisture using the “all”, “no mixed morphs”, or “singles” datasets (Table 2).

**TABLE 2:** Results of Wald Type II Anovas for the effects of morph, leaf litter treatment, and their interaction on soil temperature, soil moisture, and body size using three datasets.

Dependent variable	Independent variable	Data set								
		All			No mixed			Singles		
		*χ* ^2^	df	*p*	*χ* ^2^	df	*p*	*χ* ^2^	df	*p*
Soil temperature	Morph (M)	0.76	1	.384	1.67	1	.195	1.15	1	.283
	Leaf litter (LL)	32.65	2	**<.001**	21.41	2	**<.001**	22.11	2	**<.001**
	M × LL	3.09	2	.213	5.22	2	.07	0.80	2	.671
Soil moisture	Morph (M)	0.67	1	.415	0.33	1	.563	0.16	1	.689
	Leaf litter (LL)	4.05	2	.133	4.54	2	.103	3.60	2	.165
	M × LL	0.01	2	.995	0.08	2	.959	0.40	2	.818
Snout‐to‐vent length	Morph (M)	0.36	1	.547	0.01	1	.916	0.01	1	.943
	Leaf litter (LL)	0.60	2	.741	0.12	2	.941	0.92	2	.631
	M × LL	2.23	2	.328	2.39	2	.303	2.27	2	.322

*Note*: See text for a description of the data sets and analyses. Bolded text indicates statistical signficance.

#### 3.1.4 Habitat selection: Testing for body size differences among leaf litter treatments and morphs

There was no significant association between salamander SVL and leaf litter treatment, or between salamander SVL and morph (Table 2).

### 3.2 Refuge‐use

#### 3.2.1 Refuge‐use

**FIGURE 4:**
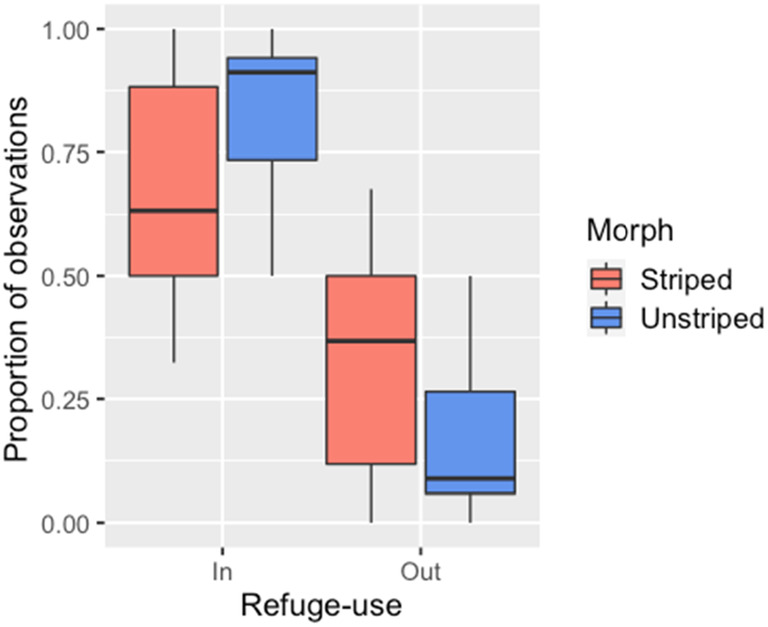
Refuge‐use by striped and unstriped adults. Salamanders scored as ‘Out’ had their head and some or all of their body out of the refuge. Each datum represents a single individual and is the proportion of observations in the behavioral category. Box and whisker plots as in Figure 3. Unstriped morphs were significantly more likely to be “In” the refuge, and striped morphs were significantly more likely to be “Out” of the refuge (see text for details).

Individual refuge‐use behavior was repeatable across trials (Pearson correlation coefficient = 0.37, *t* = 2.33, df = 34, *p* = .026). Unstriped morphs were observed in the refuge significantly more often than striped morphs (Wald *χ* ^2^ = 7.33, df = 1, *p* = .007), and striped morphs were more likely to be out of the refuge (Wald *χ* ^2^ = 5.42, df = 1, *p* = .020, Figure 4).

#### 3.2.2 Refuge‐use: Foraging success

**FIGURE 5:**
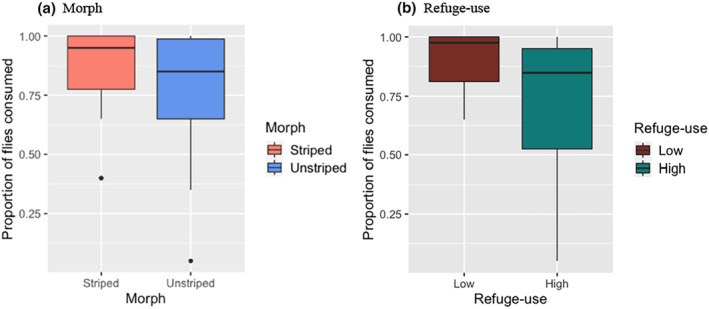
Foraging success of striped and unstriped adults (a), and of salamanders showing the lowest (*n* = 18) and highest (*n* = 18) refuge‐use (b). Box and whisker plots as in Figures 3 and 4. Each datum represents a single individual and is the proportion of flies consumed in the feeding assay. There was no significant difference between striped and unstriped morphs (a), but low refuge‐use salamanders consumed significantly more flies than high refuge‐use salamanders (see text for details).

Salamanders consumed the majority of flies that were offered (mean = 82.6%, SE = 3.88, *n* = 36). Multicollinearity between morph and refuge‐use was low enough to justify including both variables in the model (VIF = 1.2). There was no significant effect of morph or refuge‐use on foraging success (Morph: *F*_1,33_ = 0.61, *p* = .440; Refuge‐use: *F*_1,33_ = 0.79, *p* = .38, Figure 5a). However, in the categorical comparison of high versus low refuge‐use salamanders, low refuge‐use salamanders consumed a higher proportion of flies (*F*_1,34_ = 4.19, *p* = .048, Figure 5b) indicating a foraging benefit of out‐of‐refuge activity.

## 4 DISCUSSION

Unstriped adults responded positively to the leaf litter addition treatment, but striped adults did not. Thus, the unstriped morph appears to be more selective when choosing soil surface microhabitats. Leaf litter addition provided cooler soil temperatures, greater thermal buffering against changes in air temperature, and perhaps greater physical protection from predators. Our inability to detect thermal niche differences among the color morphs suggests that the morphs' differential use of the leaf litter addition treatment was not driven by differences in their thermal optima. Instead, greater thermal (and possibly hydric) stability, and/or protection from predators, may better explain the unstriped morphs' positive response to leaf litter addition. In the laboratory, unstriped morphs showed higher refuge‐use than striped morphs. These refuges provided moisture and physical protection that likely reduced salamanders' perceived risk of predation. Taken together, the field and laboratory results indicate that the unstriped morph is more sensitive to the risks of surface activity (e.g., thermal stress, dehydration, and/or predation) than the striped morph. These findings align with the view that the unstriped morph is more fossorial than the striped morph (Fisher‐Reid et al., 2013), and with the “ecomorph” model proposed by Forsman et al. (2008).

The behavioral assay showed that unstriped morphs spent more time in the refuge, and striped morphs spent more time out of the refuge. It is possible that leaving the refuge is less risky for the striped morph because of adaptations that facilitate its surface activity. More effective camouflage (Hantak & Kuchta, 2018) and a more effective tail‐autonomy response (Otaibi et al., 2017) could make the striped morph less vulnerable to predation than the unstriped morph, and unstriped morphs may compensate by increasing their refuge‐use. This kind of behavioral compensation has been observed in other prey species, e.g., small snails are more vulnerable to crayfish predators than large snails, and therefore invest more in antipredator behaviors (Dewitt et al., 1999). The refuge‐use differences we observed may also be related to morph differences in water regulation. In the laboratory assay, the refuge contained a moist paper towel, but the foraging arena did not. Striped morphs have been shown to rehydrate more quickly than unstriped morphs (Smith et al., 2015), and this physiological advantage could reduce the striped morph's risk of dehydration and facilitate out‐of‐refuge activity. It is also possible that striped morphs were more inclined to leave the refuge because they have a broader thermal niche and can seek food or mates even when the temperature is far from their thermal optima. The laboratory assay occurred at room temperature (19–21°C), which is likely above the thermal optimum of both morphs as inferred from their surface activity in the field (Anthony et al., 2008, this study). The striped morph may have evolved a broader thermal niche than the unstriped morph because it spends more time on the thermally variable surface, and less time in thermally insulated underground refuges. Finally, it is possible that striped morphs acclimate to temperature change more quickly than unstriped morphs. An ~8°C increase in temperature accompanied the transition from housing to experimental conditions in this study, and greater thermal acclimation could underly the striped morph's greater out‐of‐refuge activity. Intraspecific differences in thermal niche breadth and in thermal acclimation ability have been observed in other ectotherms (Bujan et al., 2021; Palaima & Spitze, 2004), and such differences warrant investigation in the red‐backed salamander.

This study did not reveal statistically significant differences in foraging success between the morphs in the laboratory assay. While this null result may reflect reality, limitations in the study design probably weakened our ability to detect such differences. Foraging success in the feeding assay was quite high—on average, the salamanders consumed 83% of the flies that were offered in only 1 h. Reducing the assay time, or increasing the number of flies provided, may have provided the striped morphs a greater opportunity to benefit from their higher out‐of‐refuge activity. Importantly, the categorical analysis of low versus high refuge‐use salamanders did show that out‐of‐refuge activity increased foraging success, and we interpret this result as being consistent with the hypothesis that greater surface activity would promote striped morphs' foraging success in the field. Indeed, striped morphs have been shown to obtain a more diverse and higher quality diet than unstriped morphs under field conditions (Anthony et al., 2008), although not all populations show this difference (Hantak et al., 2020). It is worth noting, however, that red‐backed salamanders do appear to forage while in underground refugia (Caldwell, 1975; Caldwell & Jones, 1973), and it would be interesting to know if the dietary differences observed between the morphs reflect, at least in part, differences in the quality and availability of above‐ and below‐ground prey.

Higher surface activity by salamanders likely promotes mating success. Previous study has revealed that striped males acquire higher quality mates than unstriped males in some populations (Acord et al., 2013; Anthony et al., 2008). In this study, striped morphs co‐occurred with the opposite sex more often than unstriped salamanders (37.3% vs. 28.9% of the time), which also suggests a striped morph mating advantage. This mating advantage could reflect differences in mate choice or, more simply, the striped morph may encounter mates more often because of its greater surface activity and/or less stringent microhabitat requirements (e.g., low or high leaf litter cover). The striped morph may pay a cost for higher mating and foraging success. At least one study has shown that striped morphs suffer greater mortality than unstriped morphs (Grant et al., 2018), perhaps because striped morphs spend more time on the soil surface where predation risk is higher. However, studies documenting the fitness benefits of being striped still outnumber those documenting the fitness costs, and studies investigating the latter are needed.

It appears that competitive interactions, in particular territoriality, had little effect on the habitat selection and refuge‐use results observed in this study. In the field, we found as many as seven individuals under a single ACO, which suggests that competition was not strong at the time of sampling. Jaeger (1979) found a similar result, with groups of 2–7 salamanders regularly occurring under cover objects in the early spring, but their clumped distribution largely disappeared by summer. Thus, it appears the effect of interference competition on red‐backed salamander spatial distributions takes time to manifest and is not apparent in the early spring when they first emerge from their underground winter retreats. This may explain why we did not find larger salamanders under the (presumably) higher quality leaf litter addition plots.

In populations where territoriality has been demonstrated, the striped morph has been shown to occupy surface territories longer and display higher levels of aggression toward potential invaders (Anthony et al., 2008; Reiter et al., 2014). If similar dynamics occur in our population, then the higher out‐of‐refuge activity shown by striped morphs in our behavioral assays could reflect greater investment in territorial defense. We investigated whether the striped morph deposited a higher proportion of its fecal pellets outside of the refuge‐tube (an indicator of territory marking) than the unstriped morph, but there was no support for this hypothesis. In addition, striped and unstriped morphs were equally likely to co‐occur with same‐sex individuals in the field. Thus, the available evidence from our study population does not point toward morph differences in territoriality, but additional work in the summer and fall seasons, especially when surface conditions are warm and dry, is needed. During these more stressful times, greater territoriality and aggression by striped morphs may force unstriped morphs underground (Dallalio et al., 2017).

Previous studies have produced conflicting results regarding thermal niche differences among the color morphs, with one finding that the unstriped morph is associated with warmer soil substrates (Moreno, 1989), one finding that the unstriped morph is associated with cooler soil substrates (Fisher‐Reid et al., 2013), and one finding that thermal niche differences among morphs vary among populations and seasons (Petruzzi et al., 2006). Hydric niche differences among morphs also vary by study, with at least one study finding unstriped morphs associated with drier soil substrates (Fisher‐Reid et al., 2013) and a second finding no evidence for hydric niche differences (Anthony et al., 2008). In this study, there was no compelling evidence for thermal or hydric niche differences among the morphs. We interpret this finding with caution, however, because the spatial and temporal scale of study may have been too limited to provide the variation in thermal and hydric conditions needed to reveal morph differences. Field observations on a larger spatiotemporal scale, combined with thermal/hydric preference tests in a controlled laboratory setting, would provide a more rigorous test of the hypothesis that color morphs in this population diverge in their thermal or hydric niches.

The behavioral niche differences observed in this study have implications for the maintenance of the polymorphism. Within a population, different behavioral strategies can provide equal fitness over the long term, enabling them to coexist (Hecht Orzack & Tuljapurkar, 2001; Mangel & Stamps, 2001). For example, in years when abiotic conditions at the soil surface are favorable, or predator populations are low, the risks of surface activity would be low and surface‐prone striped morphs may have higher fitness than unstriped morphs. However, stochasticity in environmental conditions may inevitably result in years when the unstriped morph's greater proclivity for subsurface microhabitats yields higher survival and fitness. In this way, behavioral niche partitioning and fluctuating selection could act to maintain the polymorphism (Dingemanse et al., 2004; Sih et al., 2004).

Importantly, leaf litter may play a key role in moderating differential selection between the morphs. The importance of leaf litter on the forest floor for creating a protective habitat for salamanders is well established, and reductions in leaf litter depth have been shown to negatively affect red‐backed salamander populations (Maerz et al., 2009; Ziemba et al., 2016). At first glance, the stronger response of unstriped morphs to leaf litter addition in this study might be interpreted as evidence that increasing leaf litter would favor unstriped morphs. However, if unstriped morphs spend more time inactive in protective refuges, or are generally more fossorial, then they should be less reliant on the protective effects that the leaf litter layer provides. Thus, we suggest that heavy leaf litter accumulation may favor the surface‐prone striped morph by reducing the costs of surface activity, and light leaf litter accumulation may favor the surface‐adverse unstriped morph by enhancing the costs of surface activity. Spatial variation in forest cover has been correlated with striped morph frequencies, providing indirect evidence for this hypothesis (Cosentino et al., 2017). Temporal variation in leaf litter accumulation may be equally important. For example, leaf litter accumulation on the forest floor varies seasonally, with greater leaf litter depth during the red‐backed salamanders' spring activity period than during its fall activity period (Walton, 2013). Thus, it is possible that striped morphs are favored in the spring, when the risks of surface activity are lower, and the unstriped morphs are favored in the fall when the risks of surface activity are higher. In addition, higher levels of atmospheric CO_2_, warmer temperatures, and longer growing seasons have promoted the growth of Eastern US forests (Loehle, 2020; McMahon et al., 2010). If leaf litter and woody debris accumulation on the forest floor have also increased, it could help to explain why a recent study found that striped morph frequency has increased over time (Hantak et al., 2021).

In conclusion, the behavioral differences observed in this study suggest that the unstriped morph functions as soil surface specialist, only occupying this microhabitat under ideal conditions, while the striped morph may more of a generalist, occupying the soil surface under a broader range of conditions. We suggest that these differences in microhabitat use may be important to understanding how the polymorphism is maintained. Studies that further investigate (1) morph differences in surface/subsurface activity, (2) explore the proximate bases for these differences, and (3) attempt to link morph fitness and frequencies to spatiotemporal variation in leaf litter/woody debris accumulation, provide an exciting opportunity to advance our understanding of the ecology and evolution of the red‐backed salamander.

## AUTHOR CONTRIBUTIONS

**Cory S. Straub:** Conceptualization (lead); data curation (lead); formal analysis (lead); funding acquisition (lead); investigation (lead); methodology (lead); project administration (lead); resources (lead); supervision (lead); writing – original draft (lead); writing – review and editing (lead). **Rosella G. Cuomo:** Investigation (equal); writing – review and editing (supporting). **Gabriel Jimenez:** Investigation (supporting); writing – review and editing (supporting).

## CONFLICT OF INTEREST STATEMENT

The authors declare no competing interests.

## Data Availability

Data are deposited in the Dryad data repository: doi:10.5061/dryad.tmpg4f55k.
